# Secondary Structures of Proteins Follow Menzerath–Altmann Law

**DOI:** 10.3390/ijms23031569

**Published:** 2022-01-29

**Authors:** Vladimír Matlach, Daniel Dostál, Marian Novotný

**Affiliations:** 1 Department of General Linguistics, Palacky University, 771 00 Olomouc, Czech Republic; 2 Department of Psychology, Palacky University, 771 00 Olomouc, Czech Republic; 3 Department of Cell Biology, Charles University, 128 43 Prague 2, Czech Republic; marian.novotny@natur.cuni.cz

**Keywords:** Menzerath–Altmann law, secondary structures, proteins, quantitative linguistics, empirical law, formula fitting

## Abstract

This article examines the presence of the empirical tendency known as the Menzerath–Altmann Law (MAL) on protein secondary structures. MAL is related to optimization principles observed in natural languages and in genetic information on chromosomes or protein domains. The presence of MAL is examined on a non-redundant dataset of 4728 proteins by verifying significant, negative correlations and testing classical and newly proposed formulas by fitting the observed trend. We conclude that the lengths of secondary structures are specifically dependent on their number inside the protein sequence, while possibly reflecting the formula proposed in this paper. This behavior is observed on average but is individually avoidable and possibly driven by a latent cost function. The data suggest that MAL could provide a useful guiding principle in protein design.

## 1. Introduction

The Menzerath–Altmann law (MAL) is a specific empirical relation holding between the average lengths of so-called *components* and their *constructs*. This relation was first observed on natural languages [1,2], where we find the longer words are on average (measured in syllables), the shorter are the syllables (measured in phonemes), yielding an inverse trend relation that can be described by a specific mathematical formula. Subsequently, the MAL has been observed to hold for genetic information: on genomes (constructs) and chromosomes (constituents) [3,4,5,6,7]; genes (construct) and exons (constituents) [8]; proteins (constituents) and proteomes (constructs) [9]; and, finally, proteins (constructs) and domains (constituents) [10]. The presence of the MAL is assumed to be related to the principle of least effort or the search for a balance between conflicting demands [2]. From this point of view, the MAL has been discussed as a state of equilibrium between cost (in terms of energy) and robustness and flexibility of the molecular system [10].

The purpose of this work is to assess the presence of the MAL on the secondary structures of proteins, i.e., to study whether and how the average lengths of α-helices and β-sheets (measured in the number of amino acids) are dependent on their count inside the proteins and what formula can describe this relation. This has not been yet studied; however, findings may provide information on protein design, protein evolution, protein pathology and/or protein model assessment.

The observation of the MAL in [3] raised a discussion about the inevitability or triviality of the inverse dependency described by the MAL, as it also emerged from stochastic simulations in [4]. Its inevitability has been rejected by empirical observations in [5,6,7], which point out that the MAL is at least *optional* as several species violated this inverse relation at the levels of chromosome and genomes by displaying its opposite: the longer the chromosomes, the longer the genomes. The question of inevitability based on stochastic simulations was also discussed and rejected in [11]. More recently, [12] examined the emergence of the inverse trend on language-like patterns, while pointing to the need to differentiate between two distinct principles: the Menzerath Law (ML) and the Menzerath–Altmann Law, which differ by means of its detection and implications.

ML is a label only for the presence of the inverse relation itself. It is tested by the presence of significant, negative correlations in the data (i.e., [3,4,5,8]); this inverse relation may, however, arise also from stochastic processes, and its mere presence is insufficient to draw any conclusions.

Menzerath–Altmann Law (MAL) is, in contrast to ML, a specific description of the relation defined by a specific formula. In other words, while ML assumes any, even chaotical downward trend to be accepted, the MAL assumes the existence of a specific non-chaotical average behavior reflecting a specific formula. The formulas describing the MAL were originally designed for natural languages and are examined by means of data fitting (i.e., [6,7,9,10]). It has been shown that such formulas fit natural language texts better than they can fit the products of stochastic processes.

As an implication, this article first tests the necessary ML (i.e., the inverse dependency of the average lengths of secondary structures to their number in protein) and, in the case the ML is not rejected, the work examines which specific MAL formula best describes the relation found. The formulas used are presented in [2,4,13] and include a newly developed formula. The main conclusions of this work aim to demonstrate whether an inverse relation holds between the number of secondary structures and their average lengths in the proteins, determine how this relation can be mathematically modeled, and determine what the possible implications and applications of our findings are.

## 2. Results

**Figure 1:**
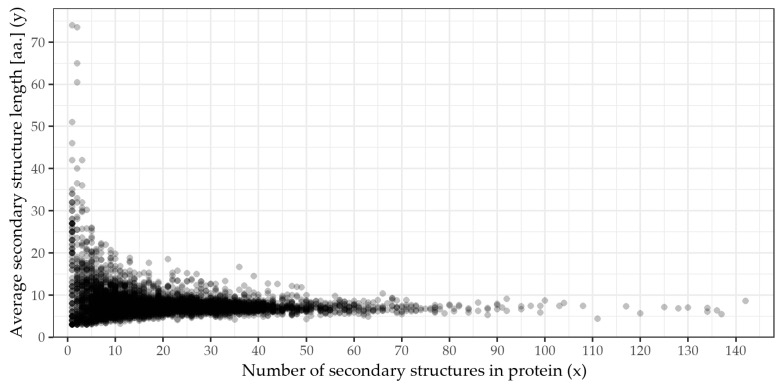
A plot of the nonredundant proteins database of 4728 proteins with experimentally determined 3D structures and continuously observed at least from 95%. Each protein of the dataset is represented by a single point. Axis *x* is the count of the secondary structures of the protein, and axis *y* is the average length of its secondary structures counted in amino acids. For example, point [*x* = 3, *y* = 21.33] stands for a protein that has three secondary structures, e.g., of lengths 29, 15 and 20 amino acids, yielding a mean average of 21.3 amino acids. Menzerath’s Law assumes there to be a downwards trend (i.e., *x* and *y* are negatively correlated), whereas the Menzerath–Altmann’s Law assumes there is a clear average trend that can be described by a specific mathematical formula. However, from this plot, it is hard to determine whether any specific correlation holds for these data, and subsequent formal analyses are required.

We collected a dataset of 4728 proteins. Each protein has an experimentally solved 3D structure continuously covering at least 95% of the sequence in order to acquire information about the number of secondary structures and their lengths (counted in amino acids). Minimum and maximum counts of secondary structures (per protein) are 1 and 142, respectively. The minimum and maximum lengths of the proteins are 7 and 2127 amino acids, respectively. To overview the dataset and its individual proteins, see Figure 1 displaying each protein as a single point. The *x* axis corresponds to the number of secondary structures of the protein, and the *y* axis is the mean average length of the protein’s secondary structures (in amino acids). Besides the expected narrowing of the *y* values as *x* increases and a potentially inverse relation between *x* and *y*, no clear trend is visible.

### 2.1. Verifying the x-y Dependence and ML

Based on [1,2,12], the next step is to verify the relation between the number of secondary structures (*x*) and their average lengths (*y*). Consequently, if this relation is verified, it then becomes necessary to verify its inverse nature—i.e., to determine whether the statement the more secondary structures, the shorter they are applies to the data.

Regarding the definition of the MAL, the test must be also applied to proteins grouped into bins (i.e., ‘binned’ data). Bins are formed by proteins with the same number of secondary structures (sharing the same *x* value). Bins have their own new *y* values calculated as an average length of all secondary structures of proteins in the bin, marked as $\bar{y}$. The bins’ minimum and maximum $\bar{y}$ values are 4.4 and 15.6 amino acids, respectively.

The results for testing the existence of the relation based on correlation tests both for the original and the binned data are presented in Table 1. The correlation coefficients are supplemented with 95% confidence intervals and *p*-values. The results show that the dependency between the average length of the secondary structures and their count is statistically significant since η (as assumed in [14]) and also Pearson and Spearman’s coefficients are significant with *p*-values < 0.001. The relation is also negative (inverse) as Pearson and Spearman’s coefficients are both negative in their whole 95%. The statement the more secondary structures, the shorter they are, applies for both original individual proteins data and their binned representation. The ML is thus not rejected for the data.

### 2.2. MAL Formula Fitting and Assessment

Since ML cannot be rejected, the presence of the MAL and the discussed formulae can be assessed on the dataset. The specific MAL formulas are listed as (1–5) below. Formulae (1–2) are originally proposed by [2]. Formula (3) is the *triviality* indicating power-law formula based on [4]. Formula (4) is its generalization where a new parameter *d*, which stands for a minimal secondary structure length, is added in analogy to [13]. Formula (5) is a newly developed empirical formula derived from the dataset by a symbol regression method for purposes of comparison, as it should fit the data better than the others. Regarding [2,6,12], we fit the formulae on the binned data obtained in the previous step, i.e., fitting *x* (the number of secondary proteins) and $\bar{y}$ (the average lengths of the secondary structures of the proteins in the bin):

(1) $$\bar{y}=ax^{b}e^{cx},$$

(2) $$\bar{y}=ax^{b},$$

(3) $$\bar{y}=ax^{-1},\;$$

(4) $$\bar{y}=d+ax^{-1},$$

(5) $$\bar{y}=\sqrt{d+ax^{-1}\;},$$

where a, b, c, d∈ℝ are model parameters that will be found by fitting the dataset. Thus, these formulae take a count of secondary structures of a protein x and calculate the expected length of the secondary structures (counted in amino acids) $\bar{y}$ and vice versa. If there is a shared, average trend in the data, it should presumably follow one of the formulae.

As noted above, Formula (2) is a general case of Formula (3), where parameter b=−1 is fixed. Thus, Formula (3) will be omitted from the graphs while its presence is assessed by Formula (2) by the non-significant difference of parameter b from −1. Next, we proceed to fit the formulae on the dataset.

The numerical results for the fits yielding final models for weighted data are presented in Table 2 with the values of the individual parameters, their standard errors and two fit quality indicators: residual standard error (*s*) and Akaike Information Criterion (AIC).

The first piece of information we gain from Table 2 is all the parameters *a*–*d* are significantly different from zero, meaning they have significant roles in their formulas and cannot be omitted without changing the quality of the model. Regarding the note above, this also applies to Formula (4) and its parameter *d*, rejecting the triviality nature of the pure power relationship attributed to the MAL in [4]. The *inevitability* is however still an open question, as Formula (4) is a generalization of (2).

The second piece of information we gain from Table 2 pertains to the quality of the individual fits. For both indicators, the residual standard error (*s*) and Akaike Information Criterion (AIC), the lower the number, the better the fit. First, using *s* as the criterion, we order the formulae from the worst to the best as follows: (3), (2), (1), (4), (5). The pure power Formula (3) is identified as the worst, causing the largest residual errors; on the contrary, its generalization (4) is the second-best. The best fit is provided by the newly proposed Formula (5). The same ordering of the formulae is also obtained by using AIC as the criterion, marking (5) as the best fitting formula. Such findings contradict the inevitability of the pure power law.

To accompany the findings, the formulas are also fitted on the non-weighted data. This step should introduce more variability in the fits and cause larger errors as the single observations may *bend* the fitted curves towards themselves more than they would if we were using the weighted data. See Table A1 in Appendix A for the fit results. Assessing the parameters, we again find that all parameters have significant roles and also that the individual formulas are again ordered (from the worst to the best scoring) as (3), (2), (1), (4), (5) for the indicator *s* and (3), (1), (2), (4), (5) for the indicator AIC. This indicates that Formulae (4) and (5) have the best fit even when encountered with more variable data.

**Figure 2:**
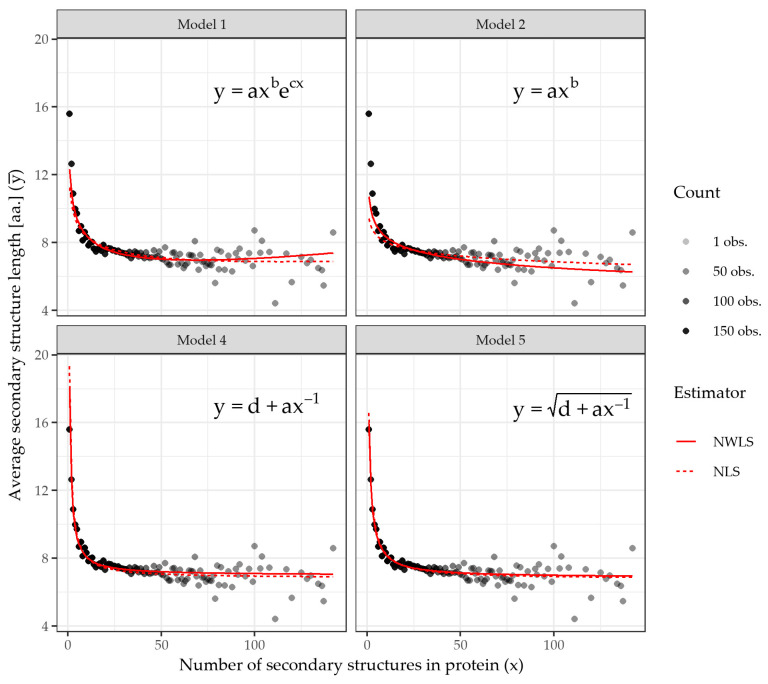
The visualization of the resulting fits of the individual Menzerath–Altmann Law formulae (Models 1, 2, 4, 5) on a dataset of 4728 proteins, binned by their number of secondary structures (i.e., the same value on the *x* axis) for the coefficients listed in Table 2. The *y* axis stands for the mean average secondary structure lengths (in amino acids) of the proteins inside the bin. The formulas are fitted by the non-linear least-squares method, reflecting weights of the individual observations counts for each bin (solid red line) and the number of secondary structures and not reflecting any weights (dashed red line). The average trend of the secondary structure lengths and counts is captured by all the models. Models 4–5 tend to have less difference on weighted and unweighted fits, showing more robustness. Let us note that Model Formula (3) as a sub-formula of (2) has a greater possibility of better fitting the data. The model predictions are also made for $x\in {\mathbb{N}}^{+}$, and as it is noticeable, Models (1) and (2) miss predicting the first upper bins (from the left) entirely. Additionally, the average trend is now clearly visible in opposition to Figure 1.

Figure 2 plots the *x* and $\bar{y}$ binned data and the individual formula fits (models), both using (solid red lines) and not using (red dashed lines) data weights. In contrast to Figure 1, the protein bins are now plotted as single points. In addition, contrary to Figure 1, a hidden average trend can be easily observed. Qualitative differences between the Models (1–5) are also noticeable, mainly at the very beginning where Models (1–2) cannot reach the upper bins. In comparison to Models (1–2) (and 3, respectively), Model (4) and especially Model (5) barely change their fit when provided with weighted or unweighted data. This observation suggests that providing weights contributes to model precision rather than bending the curve entirely. This behavior indicates that the proposed Formula (5) is more stable than the others. Let us remember Formula (3) is omitted from the plot as it is a sub-formula of (4).

Such findings imply that we can reasonably model the relation between the average lengths of secondary structures of proteins ($\bar{y}$, in amino acids) and their count inside the proteins (*x*) by means of a mathematical formula. This observation allows us to predict, interpolate, or extrapolate values for *x* and *y* for yet unseen proteins with a predefined accuracy.

### 2.3. Assessment of MAL Model Outliers

Model (5) seems like a reasonable choice for the data, although some bins deviate around the model naturally. However, larger than expected deviations are present at the individual protein level. Such unexpected deviations are of interest as they violate the formula-based balance of the average secondary structure lengths and counts. As such, proteins violating the trend fall into two categories of so-called outliers depending on whether they surpass or fall below the model.

First, we identify the surpassing protein outliers. Exactly 100 proteins belong in this group, see Appendix B for the complete list. The top extreme outliers include ATP synthase subunit b, chloroplastic (ATPF_SPIOL), Tropomyosin alpha-1 chain (TPM1_RABIT) or Cell division protein ZapB (ZAPB_ECOLI), i.e., proteins related to ATPase, muscle filament and cell division functions. However, examining outliers individually is a dangerous process as some of the outliers’ striking features may be, in fact, common in the dataset and thus expected in a random sample. A bulk approach with statistical verification is consequently used for a statistical overview of protein subcellular localizations, as listed in the UniProt hierarchy.

By assessing the subcellular localization of the surpassing outliers, we find several significant differences in how the localization should be present if the outliers were randomly sampled from the dataset (i.e., select without any rules or system). Table 3 lists such differently occurring locations within the outliers, their overall count (frequency) inside the dataset and their count among the outliers; the most extreme protein cases are provided as examples. The results show that the significant bias is towards the membrane locations (see Discussion further). For the complete list of the surpassing outliers along with the localization assessment, see Appendix B.

Second, we identify outliers that are placed unexpectedly far below the model. However, we find no such outliers in this case. This is due to the symmetrical outlier detection mechanism, which reflects mainly the more extreme surpassing proteins to which, in comparison, the proteins below are not recognizable as outliers. To obtain a few examples of the below-placed proteins, the outlier threshold is increased to −2 of the studentized standard deviations, creating an asymmetric condition. This increase provides seven outliers: Complement factor H (CFAH_HUMAN), V(D)J recombination-activating protein 2 (RAG2_MOUSE), 50S ribosomal protein L2 (RL2_DEIRA), Fascin (FSCN1_HUMAN), DNA-directed RNA polymerase II subunit RPB2 (RPB2_YEAST), Streptogramin A acetyltransferase (VATD_ENTFC) and Wound-induced proteinase inhibitor 2 (IP21_SOLLC); however, analysis of their subcellular location biases does not reveal any significant results.

## 3. Discussion

This article examined whether there exists a dependency (a correlation) between the average lengths of the secondary structures of proteins (measured in amino acids) and their counts inside the proteins. Consequently, the article examined how this relationship could be described by means of mathematical formulae known from the analogical phenomenon in natural languages. For purposes of the analysis, a nonredundant dataset of 4728 proteins with determined 3D information available for at least 95% of the protein sequences was examined.

### 3.1. Presence of Menzerath’s Law, Menzerath–Altmann’s Law and the Formulae

First, we identified that Menzerath’s Law holds for the proteins dataset, as more secondary structures led to average shorter lengths and vice versa. Consequently, the best way to describe this relation formally was assessed through Formulae (1)–(4), which were derived by theory and Formula (5), which was derived empirically from the data. The results in Table 2 (visualized in Figure 2) show the models based on Formulae (4) and (5) are more suitable than the others, and the newly proposed Formula (5) shows the best fit. Formulae (4) and (5) could be understood as a description of the average tendency of the proteins to maintain a specific—possibly optimal—ratio between their secondary structures’ lengths and their counts. As observed, this ratio can be skewed if needed as the model outliers showed.

### 3.2. Outliers of Menzerath–Altmann’s Law

The preliminary analysis of the surpassing outliers showed a systematical bias towards certain subcellular locations, mainly the cell membrane. In other words, membrane proteins violate the relation that *the more secondary structures, the shorter they are* the most from the whole dataset. The reason for this can be attributed to their nature: membrane proteins are usually composed of helical structures, which need to be typically 20–30 amino acids long to span the entire membrane [15]. The lengths of such helices have thus their specific limit, regardless of the number of transmembrane helices. Another example can be represented by human protein α-actinin 2 (ACTN2_HUMAN), whose helices create a lattice that supports the whole structure of muscle contraction, serving as a spacer of a defined length that connects actin filaments [16]—its helices have also defined lengths.

The two largest (surpassing) outliers are both ATP synthase subunits (ATPF_SPIOL, ATPF_MYCS2), both composed of two helices (126 and 21 AA.; 111 and 19 AA.) with an average length larger than that expected by the model. Both proteins are part of a large protein complex of ATP synthase.

## 4. Materials and Methods

### 4.1. Materials

Only protein sequences with experimental evidence of the protein’s existence and with experimentally solved 3D structure(s), and therefore annotated secondary structure information, were used. First, proteins with experimental evidence were extracted from the UniProtKB database release 2021_04 [17] along with their sequence lengths.

To find out which of these sequences have experimentally solved 3D structures for a complete protein, the SIFTS database was used [18]. SIFTS provides a mapping between UniProtKB and PDBe [19] at a chain and residue level. Identifiers for proteins with continuous observation of at least 95% from the original sequence length (in amino acids, AA) were obtained (i.e., 14 AA sequence must be observed whole, for 33 AA sequence, at least 32 AAs need to be observed, for 75 AA sequence at least 72 AAs are required, etc.; *continuous* means that the unobserved residues were only allowed for the very beginnings or ends of the sequences).

**Figure 3:**
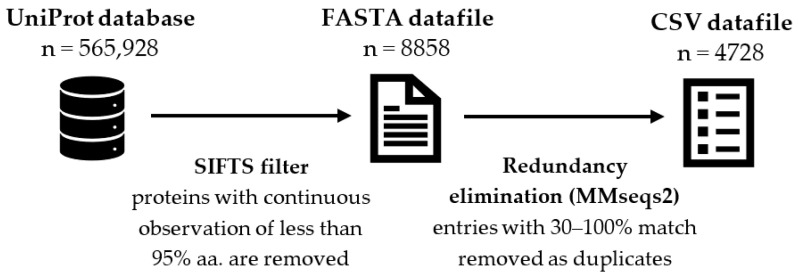
Schematic overview of the data acquisition and preprocessing. The first step is to obtain the UniProt database of reviewed proteins, where only proteins with experimental evidence are chosen for analysis. The second step is to use SIFTS database to acquire only such proteins that have experimentally solved 3D structure and at least 95% of its sequence has been observed. The third step is to use sequence clustering to obtain only proteins dissimilar to others with a threshold of 30%. The resulting dataset containing 4728 proteins is then used in this article for the evaluation of Menzerath’s Law and Menzerath–Altmann’s Law. The variable *n* stands for the number of proteins available at a given step.

To remove closely related and similar sequences from the dataset, sequences were clustered based on identity of 30% or more with MMseqs2 software [20] used, e.g., in [21,22,23,24]. (Results from methods CD-HIT, PISCES [25,26] as well as clustering levels 30% and 90% were also tested; these results lead to the same conclusions as described above.) One representative of each cluster was used to create the final list (see Appendix B for the complete list). For all the sequences in the final list, we extracted secondary structure annotations (types and lengths of secondary structure elements) and subcellular localization from UniProtKB. For the process overview, see Figure 3.

### 4.2. Methods of Testing ML and MAL

The relationship between the secondary structure counts and their average lengths counted in AA presented in Table 1 was quantified using Pearson product-moment correlation coefficient (r) and Spearman’s rank correlation coefficient (ρ). Calculations were performed on individual proteins (i.e., each data point represents one protein) as well as on groups of proteins with the same number of secondary structures (each data point corresponds to the average length of the secondary structures in a particular group; labeled as binned data). In addition to these usual indicators of linear or monotonic dependence, the correlation ratio η was also employed. This later ratio is a measure of the strength of the relationship between two variables, which does not necessarily follow a linear or (more generally) monotonic relation. This indicator is based on the sum of squares in the context of analysis of variance, and its squared value can be computed as the ratio of the between-group sum of squares to the total sum of squares.

Binned data were used in fitting statistical Models 1 to 5. Parameters of the models and their standard errors were estimated using the nonlinear weighted least-squares (NWLS) method with the Gauss–Newton algorithm. Unlike ordinary least squares (OLS), NLS (or NWLS) is an efficient tool to estimate parameters even for models that cannot be written as a linear combination of independent variables [27], as in the cases of the assessed formulas. NLS methods were used for fitting the data, e.g., in [28,29,30]. The weights were defined as w=n×x, where *n* denotes the number of proteins of a given length and *x* their length in number of secondary structures. The weights determined in this way are therefore proportional to the inverse of the sampling variance of the data points in the binned data. The models were also fitted using the nonlinear least-squares (NLS) method that applies the same weight to all data points regardless of how many and what observations they contain.

The fit of the models to the data was assessed using two indices: the residual standard error and the AIC. The residual standard error was calculated as (6):

(6) $$s=\sqrt{\frac{\sum_{j=1}^{k}w_{j\;}{\left({\bar{y}}_{j}-{\hat{y}}_{j}\right)}^{2}\;}{k-p}}\;,$$

where k∈ℕ  denotes the number of the bins, $\bar{y}\in {\mathbb{R}}^{+}$ is the average value of the length of secondary structures in the given category, $\hat{y}\in \mathbb{R}\;$ is the predicted value according to the corresponding model, and *p* ∈ℕ is the number of free parameters of this model.

The AIC is based on information theory and quantifies the amount of information contained in the data that the model is unable to reproduce. The AIC can take both positive and negative values, and in general, when comparing two models using the same dataset, the model that produces the lower AIC value exhibits a better fit [31,32]. AIC is defined as (7):

(7) AIC=2p−log(L) ,

where *p* ∈ℕ is the number of free parameters of the model, and *L* is the maximum value of the likelihood function of the model on the given data.

Some authors [33] linearize the relation by means of the logarithmic transformation of both sides of the equation before estimating the parameters. We have omitted this step since Models (4) and (5) cannot be converted to the linear form in this way. However, performing this step would produce little change in the results, and the order of magnitudes of residual standard errors does not change.

The identification of outliers is based on standardized (internally studentized) residuals. The standardized residual is the quotient obtained by dividing the raw residual by the estimate of its standard deviation. This measure of the difference between the expected and observed values makes it possible to compare the distances of individual observations from the regression curve, regardless of their weight and position on the *x*-axis. A protein with studentized standard deviations (st. res.) >3 is considered as an outlier. For the proteins below, the model threshold is increased to −2.

The statistical assessment of the occurrence of a given feature (location) among the outliers in comparison to the whole dataset is based on the UniProt annotation. The localizations contained in the dataset have 211 distinct categories. The statistical verification of the significant presence of the feature in outliers in comparison to the whole dataset is carried by hypergeometric distribution, from which the exact *p*-value can be calculated by (8):

(8) p(X≥x)∑k=x n(Mk)(N−Mn−k)(Nn) 

where x∈ℕ is a number of outliers with the examined feature, n∈ℕ is a number of all outliers, M∈ℕ is a number of proteins with the examined feature in the whole dataset, and N∈ℕ is a number of all proteins in the dataset. Consequently, since there are multiple categories, the *p*-value threshold α  = 0.05 must be corrected to $\alpha^{\prime }$ accordingly by Bonferroni Correction (9):

(9) $$\alpha^{\prime }=\frac{\alpha }{c}$$

where α∈ℝ  is the original *p*-value threshold, and c∈ℕ  is the number of categories, in our case yielding *p*-value threshold for localization $\alpha^{\prime }$ = 0.05/211 = 0.0002369668. Let us also note the Bonferroni Correction is considered conservative.

The empirical Formula (5) has been developed by symbolic regression, presented, e.g., in applications to material science in [34], to reduce mean square error (MSE; 10) of predicting $\bar{y}$ value from x on the binned protein dataset, i.e., finding a function $\bar{y}=f\left(x\right)$.

(10) $$MSE=\frac{1}{n}\sum {\left(\hat{y}-\bar{y}\right)}^{2}$$

All statistical analyses were performed using R Statistical Software (version 4.0.2). Model parameters were estimated using the *nls* function of the *stats* package.

## 5. Conclusions

### 5.1. The Average Secondary Structures Length of a Protein Is Dependent on Their Number

The results show the average lengths of the α-helix and β-sheet secondary structures measured in a number of amino acids are related to their count inside a protein and that the relation can be described by a specific mathematical formula listed as (5).

### 5.2. The Formula Describes a Possible OPTIMAL Relation

Formula (5) describes a trend followed by the average proteins, around which the others deviate. This formula is, however, derived from the data and yet lacks theoretical rationale. From this point, Formula (4), which is similar by its nature, provides a theoretical background stemming from natural language [13]. Such a formula can be understood as possibly describing the optimal relation of how many amino acids on average are used for the secondary structures when a given number of secondary structures are needed.

### 5.3. Proteins Can Outlie the Described Relation

As the results showed, proteins can outlie the average relation either by surpassing the expected average or by falling below. Such outlying can be connected with proteins residing at specific locations or having specific functions, especially membrane proteins or proteins forming large complexes (e.g., ribosome). As pointed out in the Discussion, there exist structural reasons for the membrane proteins to outlie the average relation as they need to reflect the size of the cell membranes.

### 5.4. The Relation Can Be Connected to Evolution

The observed behavior is based on the average of proteins and, as discussed above, can be avoided to some extent. This raises a question on the evolutionary perspective of proteins’ compliance with the MAL, whether balancing the number of secondary structures and their lengths yields an evolutionary advantage. In such a case, the outliers also have an evolutionary reason to avoid it, as in the case of membrane proteins, which need to reflect the size of predefined cell membranes.

### 5.5. Implications and Possible Applications

Data show the presence of MAL at the secondary structures and protein level as a possible optimal ratio between the secondary structure lengths in amino acids and their number. This can be taken into account, e.g., while designing protein sequences.

### 5.6. Further Research

We will briefly present eight possible avenues for future inquiry that stem from our investigation. First, the role of the arithmetic means in the MAL can be examined and interchanged with the trimmed mean and/or the median to examine the individual protein outliers. Second, the fit performance of various protein types/families/taxonomies can be examined and compared. Third, the role of protein domains can be examined. Fourth, a thorough analysis of MAL compliance based on protein locations and functions can be carried. Fifth, the theoretical questions raised on the prior and posterior sequence boundary existence may be researched. Sixth, testing may be conducted about whether significant deviations may be used for protein model assessment. Seventh, further research is required to determine the theoretical foundations behind Formula (5). Eighth, the MAL compliance of Transient Secondary Structures of Intrinsically disordered proteins (IDPs) in comparison to the regular secondary structures analyzed in this article can be researched [35].

## Figures and Tables

**Table 1:** Results of the test for the presence of a negative relation between number of secondary structures and their size (i.e., Menzerath’s Law) by means of calculating correlation coefficients: Pearson’s, Spearman’s and Correlation ratio η. The coefficients are calculated for the counts of secondary structures (*x*) and their average lengths counted in amino acids (*y*) and their binned version ($\bar{y}$). The coefficients are accompanied by 95% confidence intervals to support their positions and *p*-values to support their significant difference from zero. The results show the negative trend of Menzerath’s Law is statistically significant in the data examined.

Correlation	Result	95% CI	*p*-Value
Pearson r	−0.219	[−0.241, −0.199]	<0.001
Spearman ρ	−0.172	[−0.204, −0.142]	<0.001
Correlation ratio η ^1^	0.394	[0.352, 0.449]	<0.001
(binned) Pearson r	−0.495	[−0.572, −0.428]	<0.001
(binned) Spearman ρ	−0.620	[−0.703, −0.513]	<0.001

^1^ In contrast to Pearson’s r and Spearman’s ρ, Correlation ratio η does not convey information on positive or negative relation but shows only its strength on a range from 0 to 1 where 0 means zero and 1 maximal correlation.

**Table 2:** Results of fitting the individual formulas of Menzerath–Altmann’s Law on the dataset of 4728 proteins binned by the number of secondary structures. The relation of the number of secondary structures inside proteins (*x*) and their average lengths counted in amino acids ($\bar{y}$) is carried by the weighted non-linear least-squares method. The table lists the resulting fitted formula (model) parameters *a*–*d* ∈ℝ with their respective standard errors (in brackets) and summaries of the quality of the model (a) residual standard error (*s*) and (b) Akaike Information Criterion (AIC). For purposes of both *s* and AIC, the lower the number, the better the formula fits the data. The results show that all the model parameters have significant roles (i.e., are significantly non-zero) and that the best available model is (5) following both criteria *s* and AIC.

Model	a	b	c	d	*s*	AIC
1	12.305 (±0.351)	−0.176 (±0.012)	0.003 (±0.0004)		10.072	−68,085
2	10.715 (±0.247)	−0.108 (±0.007)			11.679	−68,077
3	75.238 (±7.156)				162.313	−67,444
4	11.008 (±0.515)			6.99 (±0.037)	8.763	−68,127
5	207.738 (±10.117)			46.938 (±0.575)	8.135	−68,133

**Table 3:** Statistical analysis of the subcellular locations (according to the UniProt hierarchy) of the protein outliers whose ratio of the secondary structure lengths (counted in amino acids) to their count unexpectedly surpass the value predicted by the Menzerath–Altmann Law formula fitted on the dataset of 4728 proteins. The outliers surpass three studentized residuals and more above model. The table shows localizations that are present in the group of the surpassing outliers differently than expected in comparison to their proportion in the whole dataset. Both listed unexpected differences mean the outliers are biased towards a specific location. In the case of the current data, the surpassing outliers are in cell membrane locations more than expected, i.e., the statistical test yields *p*-value < 0.05 (respectively *p*-value < 0.0002369668 after Bonferroni Correction of α).

Protein Subcellular Location	Frequency	Frequency in Outliers	Examples
Cell membrane	168 (6.9%)	23 (23.0%)	ATPF_MYCS2, CEIA_ECOLX, COX13_THET8
Cell inner membrane	103 (4.2%)	16 (16.0%)	HPPA_THEMA, EMRD_ECOLI, MURJ_THEAB

## Data Availability

Publicly available datasets were analyzed in this study. These data can be found here: https://www.uniprot.org/downloads (accessed on 28 October 2021) and https://www.ebi.ac.uk/pdbe/docs/sifts/ (accessed on 17 December 2021). The source code for software used for data preparation and extraction (as in 4.1 Materials) is available here: https://github.com/oltkkol/mal-proteins (accessed on 22 January 2022). The fitting script is available here: https://github.com/oltkkol/mal-proteins/tree/main/fitting_script (accessed on 22 January 2022). The processed data, filters and FASTA files are available here: https://github.com/oltkkol/mal-proteins/tree/main/dataset (accessed on 22 January 2022).

## Abbreviations

AA	Amino acid(s).
AIC	Akaike Information Criterion.
CI	Confidence Interval, 95%.
MAL	Menzerath–Altmann Law.
ML	Menzerath’s Law.
MSE	Mean-Squared error.
NLS	Non-linear Least Squares.
NWLS	Non-linear Weighted Least Squares.
OLS	Ordinary Linear Squares.
r	Pearson’s correlation coefficient.
s	Standard error.
st. res.	Studentized residuals.
ρ	Spearman’s rank correlation coefficient.
η	Correlation coefficient eta.
x	The number of secondary structures in a given protein.
y	The average length of the secondary structures in a given protein, measured in number of amino acids (AA).
$\bar{y}$	The average of multiple *y* values in a *bin* (i.e., a group of proteins with the same number of secondary structures *x*).

## Appendix A

The following table (Table A1) lists the results of fitting the Formulas (1)–(5) on unweighted data as the model robustness check in comparison to the fits applied on the weighted data and its results in Table 2. Since the results are not primary for the article, the table is located in the Appendix.

**Table A1:** Results of fitting the individual formulas of the Menzerath–Altmann Law on the dataset of 4728 proteins binned by the number of secondary structures. The relation of the number of secondary structures inside proteins (*x*) and their average lengths counted in amino acids ($\bar{y}$) is carried by the unweighted non-linear least-squares method. The table lists the resulting fitted formula (model) parameters *a*–*d* ∈ℝ with their respective standard errors (in brackets) and summaries of the quality of the model (a) residual standard error s and (b) Akaike Information Criterion (AIC). For purposes of both *s* and AIC, the lower the number, the better the formula fits the data. The results show all the model parameters have significant roles (i.e., are significantly non-zero) and that the best available model is (5) in both criterions s and AIC. This table serves as a supplement for the corresponding weighted fit introduced in the Results section of this article.

Model	a	b	c	d	*s*	AIC
1	11.252 (±1.42)	−0.132 (±0.042)	0.001 (±0.001)		5.325	−39,821.1
2	9.429 (±0.671)	−0.069 (±0.017)			5.361	−39,822.6
3	150.149 (±18.233)				41.601	−39,433.5
4	12.493 (±2.891)			6.825 (±0.086)	5.286	−39,822.8
5	229.332 (±58.361)			45.681 (±1.360)	5.258	−39,823.2

## Appendix B

The whole dataset of 4.728 proteins incorporating measured values and details is available here: https://github.com/oltkkol/mal-proteins/blob/main/dataset/min95obs_mmseq_30_rich.csv (accessed on 22 January 2022). The surpassing outliers information is available here: https://github.com/oltkkol/mal-proteins/blob/main/dataset/results_surpassing_outliers.tsv (accessed on 22 January 2022). The below outliers information is available here: https://github.com/oltkkol/mal-proteins/blob/main/dataset/results_below_outliers.tsv (accessed on 22 January 2022). The surpassing outliers localization assessment is available here: https://github.com/oltkkol/mal-proteins/blob/main/dataset/results_surpassing_locations.tsv (accessed on 22 January 2022).
